# Association of three 8q24 polymorphisms with prostate cancer susceptibility: evidence from a meta-analysis with 50,854 subjects

**DOI:** 10.1038/srep12069

**Published:** 2015-07-10

**Authors:** Qiaoxin li, Xia Liu, Rui-Xi Hua, Feng Wang, Hengqing An, Wei Zhang, Jin-Hong Zhu

**Affiliations:** 1 Department of Pathology, First Affiliated Hospital, Xinjiang Medical University, 137 Liyushan Road, Urumqi, Xinjiang, 830054, China; 2 Department of Oncology, First affiliated Hospital of Sun Yat-Sen University, 58 Zhongshan Er Road, Guangzhou, Guangdong, 510080, China; 3 Department of Uorology, First Affiliated Hospital, Xinjiang Medical University, 137 Liyushan Road, Urumqi, Xinjiang, 830054, China; 4 Molecular Epidemiology Laboratory and Department of Laboratory Medicine, Harbin Medical University Cancer Hospital, 150 Haping Road, Harbin, Heilongjang, 150040, China

## Abstract

The 8q24 polymorphisms have been implicated in various cancers. Three 8q24 polymorphisms (rs1447295 C>A, rs16901979 C>A, and rs6983267 T>G) have been extensively investigated for their association with prostate cancer (PCa) susceptibility, yet conclusions are contradictory. We conducted a comprehensive meta-analysis to reevaluate the associations between those polymorphisms and PCa susceptibility, according to the latest meta-analysis guidelines (PRISMA). Eligible publications were searched from MEDLINE, EMBASE and CBM. False positive report possibility analysis was performed. We totally collected 20184 cases and 20439 controls from 20 studies for the rs1447295 C>A, 1850 cases and 2090 controls from 7 studies for the rs16901979 C>A, and 12233 cases and 7582 controls from 17 studies for the rs6983267 T>G. Overall, each of studied 8q24 polymorphisms was significantly associated with PCa risk individually. Significant associations were also observed in stratified analysis by ethnicity, source of control, and quality score. Interestingly, the effect of rs1447295 on PCa risk was observed among Caucasians and Asians, but not Africa-Americans. The effect of rs16901979 was more prominent among Africa-Americans than Asians. Likewise, rs6983267 conferred a higher Pca risk among Caucasians than Asians. Collectively, these 8q24 variant(s) may modulate PCa risk in an ethnic-specific manner.

Prostate cancer (PCa) is one of the most common non-cutaneous malignancies among men in the US, with an estimated 240,890 new cases and 33,720 deaths in 2010^1^. Although little is known about the etiology of the disease, accumulating evidence has demonstrated that genetic variations may play a crucial role in the carcinogenesis of PCa. For example, genome-wide association studies (GWASs) have identified more than 40 human PCa-predisposing variants, among which single nucleotide polymorphisms (SNPs) located in the 8q24 region were considered as promising biomarkers for PCa^2^,^3^,^4^,^5^,^6^. However, not all of those significant findings can be validated by the subsequent candidate-based studies.

Chromosomal region 8q24 has emerged recently as bona fide risk locus for multiple cancers^7^,^8^,^9^. Fine mapping and additional genome scans have identified three 8q24 regions (region 1: 128.54–128.62 Mb; region 2: 128.12–128.28 Mb; region 3: 128.47–128.54 Mb) that contain variants independently associated with PCa risk^2^,^3^,^10^,^11^. Since the 8q24 region was originally shown to confer a PCa risk in a genome wide linkage scan of 871 Icelandic men in 2006^8^, numerous association studies have been performed to extensively explore the roles of 8q24 single nucleotide polymorphisms (SNPs) in the etiology of PCa. To date, there are about 64 variants in 8q24 investigated for the association with PCa risk, and only 20 of those variants were confirmed to be PCa risk-associated SNPs. Of those PCa risk SNP, rs1447295 C>A in region 1, rs16901979 C>A in region 2, and rs6983267 T>G in region 3, have shown strong association with PCa, with respective adjusted *P* value of 4 × 10^−29^ , 1 × 10^−19^, and 1 × 10^−11^ 12. Similarly, significant associations with 8q24 polymorphisms were also identified for a wide spectrum of cancers, including cancers of the breast^13^,^14^, prostate^2^,^4^, bladder^15^, colon^16^, lung^17^, ovaries^18^, pancreas^19^, and brain^20^ among different ethnicities (Asian, Caucasian, and African of Americans). Taken together, these findings have made SNPs on 8q24 of particular interest because of their potential roles in screening strategies for high-risk individuals and discovering new therapeutic targets.

The mechanisms by which 8q24 influences the course of PCa are not yet fully understood. The 8q24 region has been described as a “gene desert” since the 600-kbp gene-poor region appears to have little or no transcriptional activity. Nevertheless, several lines of evidence has suggested that 8q24 may play an active role in PCa carcinogenesis. First, 8q24 is a highly conserved genomic region. Second, *POU5F1P1*, an important gene on 8q24, encodes a weak transcriptional activator that may contribute to carcinogenesis^21^. Third, recent studies have shown that the activity of the nearby oncogene *MYC* is associated with 8q24^4^,^22^,^23^,^24^.

Although PCa carcinogenesis has been found to be associated with hereditary background, increasing molecular epidemiology studies have presented conflicting results on the association between 8q24 SNPs and PCa risk, which may be partially attributable to various sample sizes, different genetic backgrounds, and heterogeneous inclusion criteria among studies. With this in mind, we carried out the current meta-analysis to provide a quality assessment of the association of the most frequently analyzed 8q24 SNPs (i.e., rs1447295 C>A in region1, rs16901979 C>A in region2, and rs6983267 T>G in region3) with PCa risk.

## Results

### Eligible studies

Based on the inclusion criteria, 22 eligible articles consisting of 44 studies were included in this meta-analysis^1^,^12^,^25^,^26^,^27^,^28^,^29^,^30^,^31^,^32^,^33^,^34^,^35^,^36^,^37^,^38^,^39^,^40^. The sample sizes of those studies ranged from 103 to 24454. Of those articles, 9 and 13 were categorized as high and low quality, respectively, using methods described in the Methods section. Literature search and study selection yielded 20 studies for rs1447295 C>A analysis, which were performed among Caucasians (10 studies), Asians (7 studies), and Africa-Americans (3 studies). A total of 7 eligible studies were retrieved for rs16901979 C>A analysis with 3, 3, and 1 studies conducted in these three ethnic groups, respectively. Moreover, of 17 studies eligible for rs6983267 T>G analysis, there were 9, 6, and 2 carried out in these three ethnic groups, respectively. Other details were shown in Table 1. Genotype frequency distributions of studied SNPs in all of the control populations were agreed with HWE.

### Quantitative synthesis

A total 20 eligible studies were pooled together to evaluate the association between 8q24 rs1447295 C>A and PCa risk, with 20184 cases and 20439 controls. Pooled risk estimates indicated the significant associations of rs1447295 C>A with increased PCa risk under homozygous (OR= 1.39; 95% CI= 1.01–1.91, *P *= 0.042), heterozygous (OR= 1.26, 95% CI= 1.09–1.45, *P *= 0.002), dominant models (OR= 2.46, 95% CI= 1.89–3.20, *P *= 0.000), and alleles comparison (OR= 1.23, 95% CI= 1.09–1.40, *P *= 0.001); intriguingly, the direction of the association was reversed under recessive genetic models (OR= 0.51, 95% CI= 0.39–0.67, *P *= 0.001) as shown in Table 2. Moreover, there were 7 eligible studies available for 8q24 rs16901979 C>A analysis, consisting of 1850 cases and 2090 controls. We found that carrier of AA, AC, or combined carriers of AA/AC risk genotypes had an OR of 1.71 (homozygous: 95% CI= 1.36–2.16, *P *= 0.000), 1.31 (heterozygous 95% CI= 1.12–1.53, *P *= 0.001), or 1.39 (dominant: 95% CI= 1.20–1.61, *P *= 0.000) for developing prostate cancer, respectively, compared with those with CC genotype. And OR of alleles comparison (C vs. A) for the association was 1.31 (95% CI= 1.18–1.46, *P *= 0.000). For 8q24 rs6983267 T>G analysis, a total of 17 studies comprising 12233 cases and 7582 controls were incorporated into the meta-analysis. Pooled analysis observed significant associations between increased PCa risk and the variant of interest under homozygous (OR= 1.44; 95% CI= 1.31–1.58, *P *= 0.000), heterozygous (OR= 1.19, 95% CI= 1.10–1.29, *P *= 0.000), recessive (OR= 1.26, 95% CI= 1.18–1.36, *P *= 0.000), and alleles comparison (OR= 1.19, 95% CI= 1.14–1.25, *P *= 0.000).

### Stratification analysis

In the stratification analyses, subgroups with less than three studies were excluded from further analysis because of small number of subgroups would likely lead to a false association. In the current meta-analysis, positive associations were found among most of the remaining subgroups for each of the three 8q24 SNPs. Briefly, the associations of rs1447295 C>A polymorphism and PCa risk were observed among Asians populations under the all assumed genetic comparisons, among Caucasians under recessive and dominant models, but not among Africa-Americans. Similarly, stratified analysis by source of control found that rs1447295 C>A showed significant association with PCa risk for PB subgroup under all genetic models, while the association was only detected under recessive, dominant model, and allele comparison for HB subgroup. Moreover, the associations were observed for both low and high quality subgroups.

As shown in Table 2, due to the limited number of study, stratification analysis for rs1601979 C>A polymorphism was not conducted among Caucasians and PB studies. The significant risk effects of rs1601979 C>A polymorphism on PCa risk were consistently observed under the most of assumed genetic comparisons for Africa-Americans, hospital-based studies, and studies with high quality score. The effects were also existed among Asians and studies with low quality score, with fewer genetic models detecting the significance. We then compared the strength of the associations within the subgroups, the association with larger OR and smaller *P* value was considered as more prominent than others. Interestingly, the association between PCa and rs16901979 was more prominent among Africa-Americans than Asians under most of genetic models (e.g. Africa-Americans with homozygous OR= 1.91, 95% CI= 1.44–2.55, *P *= 0.000; heterozygous OR= 1.41 (1.10–1.80), *P *= 0.000; dominant OR= 1.56, 95% CI= 1.24–1.96, *P *= 0.000; and allele comparison OR= 1.40, 95% CI= 1.21–1.62, *P *= 0.000. In contrast, Asians with recessive OR= 1.65, 95% CI= 1.13–2.41), *P *= 0.000; and allele comparison OR= 1.17, 95% CI= 1.00–1.38, *P *= 0.053) . Moreover, stratified analyses observed significantly increased PCa risk associated with rs6983267 T>G polymorphism among all subgroups defined by ethnicity, source of control, and quality score. Likewise, rs6983267 T>G seemed to confer a higher Pca risk in Caucasians than Asians (homozygous OR= 1.47, 95% CI= 1.33–1.64, *P *= 0.000 vs. OR= 1.33, 95% CI= 1.08–1.62, *P *= 0.006; Allele comparison OR= 1.21, 95% CI= 1.15–1.27, *P *= 0.000 vs. Allele comparison OR= 1.14, 95% CI= 1.03–1.25, *P *= 0.01).

**Figure 1:**
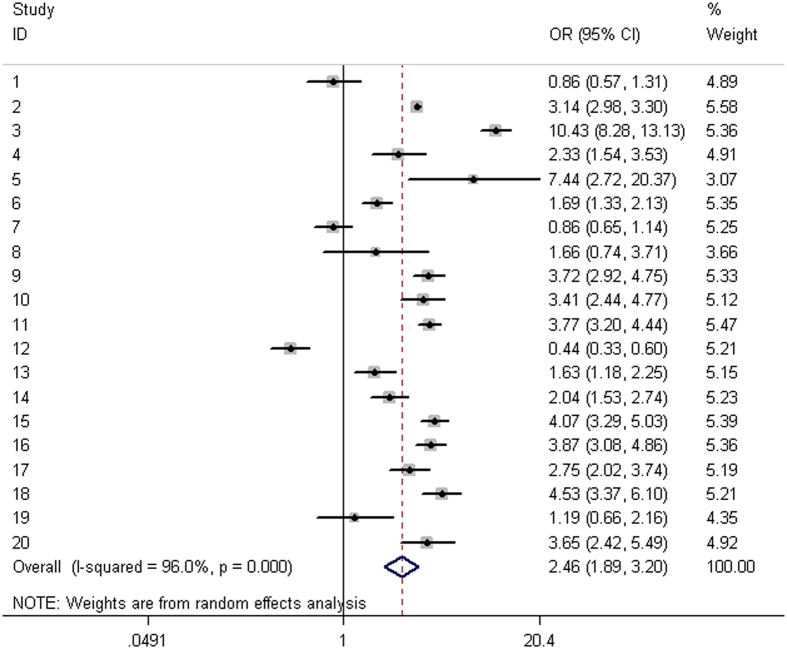
Forest plots of effect estimates for *8q24*-rs1447295 polymorphism and prostate cancer risk (AA+AC vs. CC). The size of the squares indicates the relative weight of each study. Weights were derived from random-effects analysis. Bars, 95% confidence interval (CI).

In the current meta-analysis, a high level of heterogeneity was detected in the pooled analysis for rs1447295 C>A. However, the heterogeneity decreased in the stratified analysis by ethnicity, indicating that ethnicity might be a source of heterogeneity. Moreover, the between-study heterogeneity was detected among several subgroups for each of studied SNPs. However, leave-one-out sensitivity analyses suggested that no single study substantially altered the pooled ORs of any SNP of interest (data not shown), suggesting the stability of this meta-analysis. Finally, the FPRP values for all significant findings at different prior probability levels were summarized in Table 3. With the assumption of prior probability of 0.01, the association with rs1447295 C>A was noteworthy for Asians, population-based studies and high quality studies (FPRP range: 0.010–0.144, 0.010–0.022, 0.010–0.014, respectively) under the assumed comparisons. Moreover, the association between PCa and rs16901979 C>A among Africa-American (FPRP: 0.026) under dominant and allele comparison was considered noteworthy. In term of rs6983267 T>G, the association was noteworthy for Caucasians, population-based studies, high quality studies, low quality studies. In contrast, based on FPRP values, the rest of significant associations between 8q24 variants and PCa risk might not be deserving of attention, suggesting some possible bias or chance finding in this meta-analysis. Therefore, our findings need to be further validated in large and well-designed studies, involving different ethnicities (Fig. 1).

### Publication Bias Analysis

**Figure 2:**
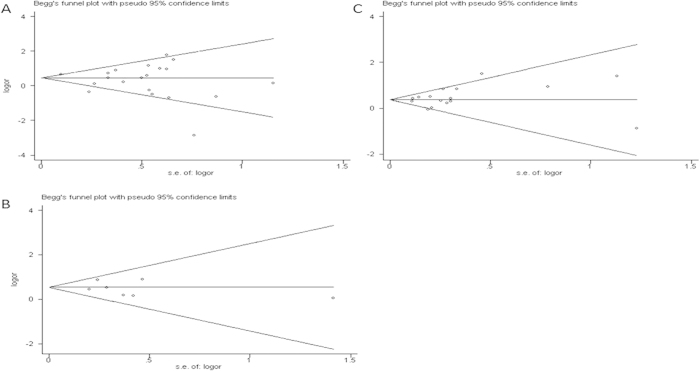
Funnel plot analysis to detect publication bias for each of the *8q24* polymorphism by homozygous model. (**A**) For rs1447295 polymorphism, (**B**) For rs16901979 polymorphism, and (**C**) For rs6983267 polymorphism. Each point represented an individual study for the indicated association.

Publication bias was examined by Begg’s funnel plots and Egger’s tests across all genetic models for each of the three polymorphisms in the 8q24 region. The shape of funnel plot (Fig. 2) was symmetrical, suggesting that there was no evidence of publication bias in the meta-analysis.

## Discussion

To the best of our knowledge, this is the largest meta-analysis to investigate the association between the selected 8q24 SNPs and PCa risk based on the candidate association studies. Up to now, more than 64 8q24SNPs have been explored for their role in PCa carcinogenesis, among which about 20 SNPs are regarded as PCa-related SNPs. However, those results were often controversial. In this meta-analysis, we evaluated the association of PCa risk with three most frequently investigated SNPs that scatter through the three regions of 8q24, respectively. We found significant associations between each of these 8q24 SNPs and PCa risk under most of the assumed comparisons, either in overall or in stratified analyses by ethnicity, source of control, and quality score. Intriguingly, stratified analyses by ethnicity revealed significant association between rs1447295 C>A and PCa risk among Caucasians and Asians, but not Africa-Americans. The effect of polymorphism rs16901979 C>A on PCa risk was more apparent in Africa-Americans populations when compared with Asian populations. Similarly, rs6983267 conferred a higher Pca risk among Caucasians than Asians. Although it is difficult to explain the ethnic-specific findings, the disparity in different ethnic populations is not completely surprising. Incidence rates of PCa vary internationally by over 25-fold. Developed countries in Oceania, Europe, and North American have the highest rates of PCa, while incidence in Asia is relatively low^41^. On the contrary, the highest prostate cancer mortality rates are recorded in African descent in the Caribbean region, which is partly explained by genetic predisposition^42^,^43^. Taken together, these epidemiological findings have suggested that both environmental and genetic factors may have profound impacts on PCa. Therefore, we speculated that differences in environments, genetic backgrounds, and gene-environment interaction may help to interpret the different results in the different ethnic groups. Future studies may provide some insights into the ethnic-specific findings, focusing on a difference in LD between these variants and the causal variants in each ethnic group, epistasis with other variants found in these ethnicities, or gene-environment interaction.

**Figure 3:**
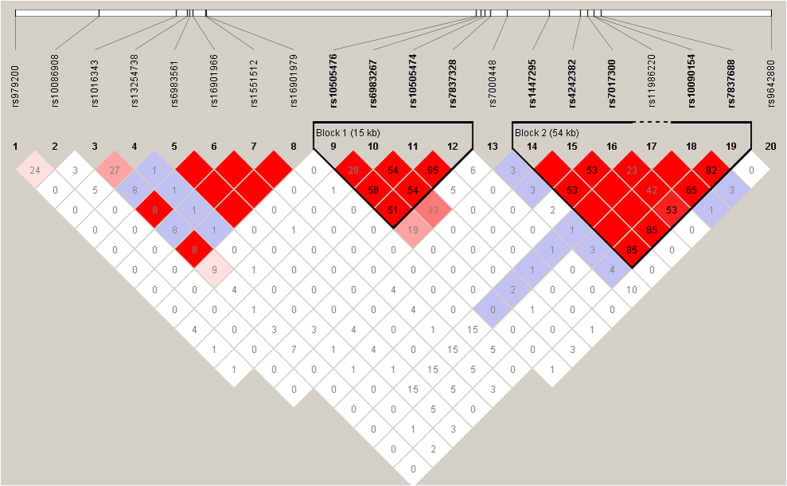
Linkage disequilibrium in the 8q24 region for the previously reported polymorphisms. Linkage disequilibrium coefficients (r^2^) between SNPs in the 8q24 region between 128.12 and 128.54 Mb.

Moreover, although mechanisms by which 8q24 modulates the risk of PCa is poorly understood, mounting evidence from genetic association studies and fine-mapping informatics supports that 8q24 genetic variant may impart susceptibility to PCa in an ethnic-specific manner. 8q24 region is commonly subdivided into three regions on the basis of the local genetic characteristics of 8q24 and the fine-mapping study^11^. Region 1 (126.54–128.62 Mb) was initially identified by the original linkage and admixture studies in Icelandic families populations; and initial association studies indicated that this region might contribute to a higher incidence of PCa in Africa-American men than men of European ancestry^7^,^8^. Region 2 (128.14–128.28 Mb) harbors a 14-SNP haplotype that efficiently tags a relatively uncommon (2–4%) susceptibility variant in individuals of European descent, which happens to be very common (42%) in Africa-Amricans^2^,^10^. And region 3 (128.47–128.54 Mb) is defined as a recombination hot-spot among European Americans. There is little linkage disequilibrium among SNPs across the three regions. Although regions 1 and 3 are physically close to each other, they are separated by a recombination hotspot among individuals of European ancestry^2^. Hence, SNPs across all three neighboring regions seem to independently influence 8q24 signal transduction, and the combined effects of SNPs across regions closely follow a multiplicative model^2^,^10^. Human chromosome 8q24 contains three regions spanning 6000 kb, according to the dbSNP database (http://www.ncbi.nlm.nih.gov/SNP), in which more than 207 variants were reported. Twenty previously reported 8q24SNPs statistically significantly associated with PCa risk show various linkage disequilibrium (LD) with a range from low to high degree (Fig. 3). These SNPs can be divided into three blocks. For example, block1 included rs698267, rs10505476, rs10505474, and rs7837328, showing LD with r^2^ values ranging from 0.28 to 0.95. Block2 consisted of rs1447295, rs4242382, rs7017300, rs11986220, rs10090154, and 7837688, with LD (r^2^) varying from 0.23 to 1.0. Additionally, rs16901979 was found in high LD of 1.0 with some polymorphisms including rs6983561, rs16901966, and rs1551512. None of the variants resides within known genes, of which there are few across the regions of 8q24. It was predicted that those risk-associated variants could affect the regulation or transcription of a causal pseudogene or gene outside the region, including proto-oncogene MYC (located approximately 260 kb telomeric to region 1), FAM84B, TCF7L2, and POU5F1^21^,^44^, but the biological mechanisms underlying these findings remain unclear. Another speculation that may help to explain the association is that some functional polymorphisms linked to those SNPs may account for the association. However, based on current understanding, none of these variants and their respectively linked-SNPs seems to be potentially functional. Therefore, the exact mechanisms underlying the observed association of the SNPs with PCa risk need more investigations.

8q24 was considered as a gene-free region, flanked by the *FAM84B* and *MYC* genes on the centromeric and telomeric ends, respectively. Although data based on epidemiologic studies have indicated the possible association between 8q24 variants and PCa risk, little is known about the mechanisms. There are significant differences in allele frequency distribution of the three selected SNPs among different ethnic-populations, indicating these 8q24 variant(s) may modulate PCa in an ethnic-specific manner. One study suggested that variants in this region may be a part of the *cis*-regulatory enhancer element for the *MYC* gene, with a long-range physical interaction with this gene in PCa^4^. For example, a recent report indicated that rs6983267 displayed an altered capacity of binding of transcription factor 7-like 2 (*TCF7L2*), thereby leading to a different physical interaction with *MYC*^44^. However, others failed to find clear association between rs6983267 genotype and MYC expression. Therefore, more powerful studies should be performed to further investigate the function of the risk- associated SNPs.

Up to now, several meta-analyses have investigated the associations between 8q24 polymorphisms and PCa risk^45^,^46^,^47^. And consistent findings were observed among the previously reported meta-analyses and the present study, indicating that 8q24-rs1447295, -rs16901979, and –rs6983267 polymorphisms might confer genetic susceptibility to PCa. Additionally, this meta-analysis was the first to further explore the associations by stratification analyses (ethnicity, source of control, and quality score), as compared to others. However, although significant associations were found in the stratification analyses by ethnicity, these findings warrant further validation in large, well-designed, multiethnic case-control studies.

Finally, some limitations in the meta-analysis should be addressed. First, relatively small samples were included for the assessment, especially for rs16901979 polymorphism. Second, the controls were not uniformly defined, which might result in obscure findings. Third, there was a significant heterogeneity in different ethnic groups; the heterogeneity may be partially attributed to the difference in individuals’ genetic backgrounds or the environment in which subjects live, since these genetic and environmental factors may modify the risk effects of studied SNP on cancer susceptibility. Fourth, due to inadequate information, we failed to control for possible confounders such as age, smoking, obesity, alcohol consumption, and other lifestyle risk factors, and our results were based on unadjusted estimates. Finally, the ethnic-specific effects of the studied SNPs on PCa risk were not adequately discussed due to lack of relevant publications.

In summary, this meta-analysis provided evidence of the association between 8q24 polymorphisms (i.e. rs1447295 C>A, rs16901979 C>A, and rs6983267 T>G) and PCa risk, suggesting that these 8q24 variants may be a low penetrance contributor to PCa. Considering that PCa is one of genetic and geographic related malignancies, more relevant SNPs, variants, SNP-induced gene structural changes, epigenetic changes, and interactions of SNP-SNP, gene-gene, and gene-environment should be addressed in future large multicentric studies, which should lead to better, comprehensive understanding of the association between the 8q24 Polymorphisms and PC risk.

## Methods

### Search strategy and selection criteria

**Figure 4:**
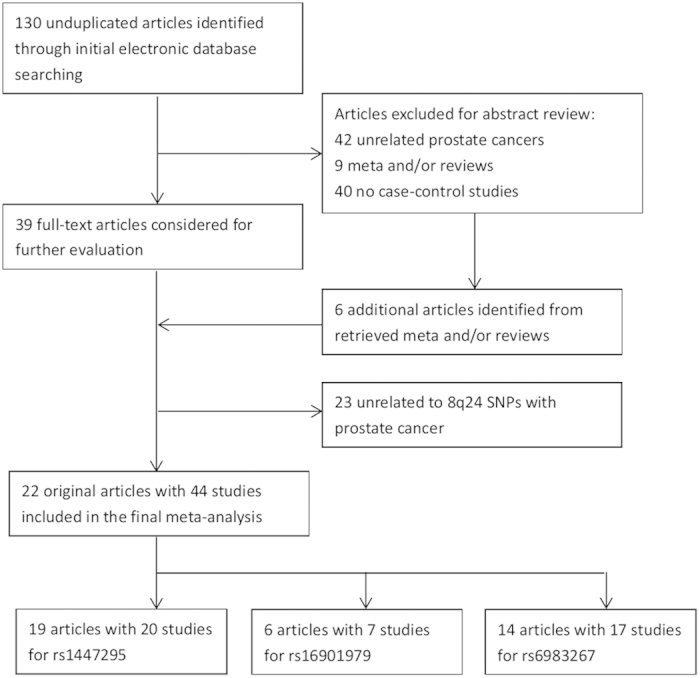
Flow chart of identification of included studies.

A comprehensive literature search was carried out for publications investigating the association between 8q24 polymorphisms and PCa risk. We searched PubMed, Embase and China Biology Medicine (CBM) databases up to June 2014 using the following keywords, including “8q24”, “variant or polymorphism”, “cancer, carcinoma, or malignancy”, and “prostate disease”. Further limited searching strategy included “English and Chinese language publications” and “human species”. Abstracts alone and unpublished articles were not considered. Moreover, the reference lists of the retrieved articles and reviews were sought manually to find relevant original articles. Eligible Studies for this meta-analysis were required to meet all of the following criteria: (i) studies on associations of at least one of 8q24 polymorphisms (rs1447295 C>A, rs6983267 T>G, rs16901979 C>A) with PCa risk; (ii) enrolled patients had sporadic, primary and histologically confirmed PCa, and controls had no history of cancer or neoplasm; (iii) case-control or nested case-control studies; (iv) availability of genotype data of both case and control group to calculate the odds ratio (OR) with its 95% confidence interval (CI) and *P* value; and (v) genotype distribution in controls is complied with the Hardy-Weinberg equilibrium (HWE, *P*>0.05). All of the included studies were candidate-based association studies. If participants in studies overlapped, only the study with the largest sample size was included in the meta-analysis. The flow chart of identification the eligible studies was shown in Fig. 4, and the protocol was checked according to the PRISMA checklist (Supporting Information).

### Assessment of study quality: Extended Quality Score

The quality of each included investigation was estimated using Extended Quality Score to limit the risk of introducing bias into meta-analyses or systematic reviews. Each article is defined as “high” or “poor” quality according to an extended-quality scale system by evaluating special characteristics of the participant groups and genotyping methods of the studies^48^. In this meta-analysis, two investigators appraised the quality of each investigation. If there was any disagreement, a third investigator (Jin-Hong Zhu) would mediate and a decision would be made by voting.

### Data extraction

Two reviewers (Qiaoxin Li and Xia Liu) independently appraised and extracted data according to the inclusion criteria listed above. Disagreements were documented and resolved by full discussion with a third reviewer (Jin-Hong Zhu) until a consensus was reached. The following information were extracted from each eligible study: the first author’s surname, year of publication, country in which study was conducted, ethnicity of participants (categorized as Caucasians, Asians, and African descents), study design, sample size, source of control, *P* value of HWE test, genotyping methods, genotype counts of case and controls for three studied SNPs, and the clinical/pathological characteristics of cases. For studies involving subjects of different ethnic groups, data were extracted and split by ethnicity.

### Statistical analysis

HWE in the control groups was assessed by the Pearson’s goodness-of-fit chi-square test, with a *P* value less than 0.05 indicating derivation from HWE, and population was regarded as suitable if more than one SNP agreed with HWE. The strength of association between 8q24 polymorphisms and PCa risk was estimated by calculating odds ratios (OR) and the corresponding 95% confidence intervals (CI). For each of studied SNP, we conducted comparison of the frequency of wild (*W*) and variant (*V*) alleles between PCa cases and controls (*V* vs. *W*). Moreover, pooled ORs were calculated under the following genetic models: homozygous (*VV* vs. *WW*), heterozygous (*VW* vs. *WW*), dominant (*VV* + *VW* vs. *WW*), and recessive (*VV* vs*. VW* + *WW*) models. Stratification analyses were performed by ethnicity, source of control, and quality score, but not by tumor stage, Gleason score, and PAS levels because of the small size of the eligible studies. For each genetic model, a χ^2^-based Q-test was performed to check the presence of between-study heterogeneity across all of the comparisons at a level of significance of 0.10^49^. In addition, the heterogeneity was also quantified with *I*^*2*^ statistics, which is independent of the number of studies included in the meta-analysis. *I*^*2*^ lies between 0% and 100% with higher values indicating a greater heterogeneity^50^. The fixed-effects model was chosen to calculate the combined OR in the absence of heterogeneity. Otherwise, a random-effects model was used to estimate the pooled OR. The significance of the pooled OR was determined using the Z test. Finally, false positive report probability (FPRP) was introduced as a multiple testing for the whole significant associations. The statistical power of the current study was calculated with an FPRP threshold of 0.2 was defined as true finding, i.e., the probability of a false positive result is <20%^49^. In sensitivity analysis, following the sequential exclusion of a single study at a time, the pooled estimates were recomputed to determine the stability of the results. Furthermore, potential publication bias was assessed by visual inspection of Begg’s funnel plots with an asymmetry plot suggested possible publication bias^51^. All statistical analyses were performed using STATA statistical software (version 11.0, StataCorp, College Station, TX) and SAS version 9.1 (SAS Institute, Cary, NC). Two-sided *P* values were adopted, and *P* <0.05 were considered statistically significant without further notification.

## Additional Information

**How to cite this article**: li, Q. *et al.* Association of three 8q24 polymorphisms with prostate cancer susceptibility: evidence from a meta-analysis with 50,854 subjects. *Sci. Rep.* **5**, 12069; doi: 10.1038/srep12069 (2015).

## Figures and Tables

**Table 1:** Characteristics of studies included in the current meta-analysis.

Surname	Year	Country	Ethnicity	Source	Genotype method	Case				control				MAF	HWE	Score
						*WW*	*VW*	*VV*	ALL	*WW*	*VW*	*VV*	ALL			
For rs1447295 C>A (region1)																
Chan	2013	Singapore	Asian	HB	Illumina 1M chip	180	92	17	289	94	44	5	143	**0.19**	0.957	7
Schumacher	2007	USA	Caucasians	PB	TaqMan	8462	2736	268	11466	10344	2472	172	12988	**0.11**	0.079	14
Zheng	2007	USA	Caucasians	HB	iPLEX	1169	346	31	1546	485	82	4	571	**0.08**	0.794	11
Joung	2012	Korea	Asian	HB	iPLEX	114	67	12	193	127	38	3	168	**0.13**	0.936	7
Francisco	2013	Chile	Caucasians	HB	TaqMan	56	23	4	83	16	4	1	21	**0.14**	0.309	4
Terada	2008	Japan	Asian	HB	PCR-RFLP	310	172	25	507	335	165	11	511	**0.18**	0.071	9
Okobia	2011	USA	Africa-American	HB	TaqMan	156	162	36	354	173	207	58	438	**0.37**	0.291	11
Brankovie	2013	Sebia	Caucasians	HB	PCR-RFLP	86	61	3	150	11	82	7	100	**0.48**	0.000	7
Suurinirmi	2007	USA	Caucasians	PB	TaqMan	435	136	11	582	427	107	4	538	**0.11**	0.390	15
Zeegers	2011	USA	Caucasians	PB	TaqMan	224	53	4	281	196	64	7	267	**0.15**	0.523	10
Salinas	2008	USA	Caucasians	PB	TaqMan	937	288	27	1252	994	225	14	1233	**0.10**	0.753	15
Benford	2010	USA	Africa-American	HB	TaqMan	86	77	26	189	237	221	65	523	**0.34**	0.000	8
Liu	2012	China	Asian	PB	PCR-RFLP	150	102	8	260	197	86	4	287	**0.16**	0.111	11
Liu	2009	Japan	Asian	HB	TaqMan	217	149	25	391	218	89	16	323	**0.19**	0.088	8
Severi	2007	Australian	Caucasians	PB	TaqMan	595	212	14	821	586	135	11	732	**0.11**	0.319	13
Wokolorczyk	2010	Poland	Caucasians	HB	PCR-RFLP	515	156	19	690	484	115	3	602	**0.10**	0.165	8
Chen	2009	Taiwan	Asian	PB	TaqMan	215	119	6	340	253	75	9	337	**0.14**	0.237	10
Cheng	2008	USA	Caucasians	HB	TaqMan	318	97	2	417	344	69	4	417	**0.09**	0.795	11
Cheng	2008	USA	Africa-American	HB	TaqMan	39	44	6	89	43	35	11	89	**0.32**	0.919	11
Zheng	2010	China	Asian	PB	iPLEX	173	96	15	284	110	35	6	151	**0.16**	0.147	12
For rs16901979 C>A (region2)																
Chan	2013	Singapore	Asian	HB	Illumina 1M chip	139	119	31	289	64	68	12	144	**0.32**	0.302	7
Joung	2012	Korea	Asian	HB	iPLEX	99	81	14	194	100	57	12	169	**0.24**	0.333	7
Okobia	2011	USA	Africa-American	HB	TaqMan	81	158	99	338	131	193	102	426	**0.47**	0.064	11
Chen	2010	Taiwan	Asian	HB	TaqMan	148	148	35	331	173	138	24	335	**0.28**	0.62	11
Benford	2010	USA	Africa-American	HB	TaqMan	45	97	50	192	188	237	87	512	**0.40**	0.406	8
Cheng	2008	USA	Caucasians	HB	TaqMan	375	41	1	417	393	22	1	416	**0.03**	0.252	11
Cheng	2008	USA	Africa-American	HB	TaqMan	23	43	23	89	27	50	11	88	**0.41**	0.1	11
For rs6983267 T>G (region3)																
Chan	2013	Singapore	Asian	HB	Illumina 1M chip	89	136	63	288	47	74	23	144	**0.42**	0.493	7
Zheng	2007	USA	Caucasians	HB	iPLEX	495	771	285	1551	142	299	132	573	**0.49**	0.293	11
Joung	2012	Korea	Asian	HB	iPLEX	56	92	46	194	51	86	31	168	**0.44**	0.618	7
Francisco	2013	Chile	Caucasians	HB	TaqMan	19	33	30	82	5	13	3	21	**0.45**	0.253	4
Terada	2008	Japan	Asian	HB	PCR-RFLP	211	219	77	507	206	225	80	511	**0.38**	0.94	9
Okobia	2011	USA	Africa-American	HB	TaqMan	307	34	2	343	373	52	1	426	**0.06**	0.562	11
Brankovie	2013	Sebia	Caucasians	HB	PCR-RFLP	53	80	17	150	69	129	52	250	**0.47**	0.561	7
Salinas	2008	USA	Caucasians	PB	PCR-RFLP	364	652	242	1258	313	617	308	1238	**0.50**	0.91	15
Liu	2012	China	Asian	PB	PCR-RFLP	70	137	53	260	94	137	51	282	**0.42**	0.93	11
Liu	2009	Japan	Asian	HB	TaqMan	151	181	59	391	147	151	25	323	**0.31**	0.104	8
Papanikolopoulou	2011	Greece	Caucasians	HB	TaqMan	16	46	24	86	39	47	13	99	**0.37**	0.844	6
Cheng	2008	USA	Caucasians	HB	TaqMan	76	215	126	417	106	206	105	417	**0.50**	0.807	11
Cheng	2008	USA	Africa-American	HB	TaqMan	74	14	1	89	74	11	4	89	**0.11**	0.001	11
Zheng	2010	China	Asian	PB	iPLEX	86	134	62	282	51	72	29	152	**0.43**	0.69	12
Penney	2009	USA	Caucasians	PB	iPLEX	400	644	261	1305	372	707	323	1402	**0.48**	0.714	15
Penney	2009	USA	Caucasians	PB	iPLEX	1184	1776	812	3772	69	134	46	249	**0.45**	0.177	15

HB, Hospital based; PB, Population based; PCR-RFLP, Polymorphism chain reaction-restriction fragment length polymorphism; MAF, Minor allele frequency; HWE, Hardy-Weinberg equilibrium. WW, wild homozygous; WV, heterozygous; VV, variant homozygous.

**Table 2:** Meta-analysis of the association between 8q24 rs1447295, rs16901979 and rs6983267 polymorphisms and prostate cancer risk.

Variables	No. of individuals	No. of studies	Homozygous		Heterozygous		Recessive			Dominant		Allele	
			OR (95% CI)	*P*^het^	OR (95% CI)	*P*^het^	OR (95% CI)	*P*^het^		OR (95% CI)	*P*^het^	OR (95% CI)	*P*^het^
rs1447295 C>A (region1)													
All	20184/20439	20	1.39 (1.01–1.91)	0.000	1.26 (1.09–1.45)	0.000	0.51 (0.39–0.67)	0.001		2.46 (1.89–3.20)	0.000	1.23 (1.09–1.40)	0.000
publication bias (Begg’s test)			*P *= 0.296		*P *= 0.382		*P *= 0.713			*P *= 0.277		*P *= 0.272	
Ethnicity													
Caucasion	17288/17469	10	1.35 (0.78–2.35)	0.000	1.15 (0.92–1.44)	0.000	0.48 (0.31–0.74)	0.014		4.15 (3.26–5.28)	0.000	1.18(0.97–1.43)	0.000
Asian	2264/1920	7	1.87 (1.33–2.62)	0.469	1.49 (1.30–1.70)	0.112	0.38 (0.27–0.53)	0.438		1.97 (1.47–2.62)	0.000	1.44 (1.29–1.60)	0.425
Africa-American	632/1050	3	0.82 (0.59–1.14)	0.349	0.95 (0.77–1.18)	0.411	0.91 (0.66–1.26)	0.992		0.74 (0.43–1.29)	0.001	0.92 (0.79–1.07)	0.487
Source of control													
HB	4898/3906	12	1.28 (0.75–2.17)	0.000	1.12 (0.84–1.49)	0.000	0.52 (0.33–0.82)	0.001		2.07 (1.16–3.67)	0.000	1.16 (0.92–1.47)	0.000
PB	15286/16533	8	1.80 (1.52–2.13)	0.278	1.38 (1.22–1.57)	0.000	0.44 (0.37–0.52)	0.329		3.22 (2.78–3.74)	0.000	1.34 (1.21–1.50)	0.049
Score													
Low	2492/2391	8	1.49 (0.74–2.99)	0.000	1.17 (0.92–1.51)	0.000	0.45 (0.25–0.81)	0.003		1.79 (1.01–3.16)	0.000	1.14 (0.82–1.58)	0.000
High	17692/18048	12	1.32 (0.92–1.91)	0.002	1.19 (0.82–1.71)	0.003	0.54 (0.39–0.74)	0.016		3.05 (2.31–4.02)	0.000	1.29 (1.14–1.46)	0.000
rs16901979 C>A (region2)													
All	1850/2090	7	1.71 (1.36–2.16)	0.595	1.31 (1.12–1.53)	0.136	1.22 (0.80–1.87)	0.002		1.39 (1.20–1.61)	0.144	1.31 (1.18–1.46)	0.232
publication bias (Begg’s test)			*P *= 0.609		*P *= 0.948		*P *= 0.995			*P *= 0.854		*P *= 0.689	
Ethnicity													
Asian	714/648	3	1.41 (0.95–2.09)	0.661	1.15 (0.92–1.43)	0.134	1.65 (1.13–2.41)	0.293		1.19 (0.97–1.47)	0.179	1.17 (1.00–1.38)	0.350
Africa-American	619/1026	3	1.91 (1.44–2.55)	0.342	1.41 (1.10–1.80)	0.385	0.96 (0.54–1.71)	0.007		1.56 (1.24–1.96)	0.413	1.40 (1.21–1.62)	0.415
Source of control													
HB	1850/2090	7	1.71 (1.36–2.16)	0.595	1.31 (1.12–1.53)	0.136	1.22 (0.80–1.87)	0.002		1.39 (1.20–1.61)	0.144	1.31 (1.18–1.46)	0.232
Score													
Low	675/825	3	1.75 (1.22–2.51)	0.160	1.26 (0.8–1.97)	0.032	1.49 (1.08–2.05)	0.475		1.32 (0.84–2.08)	0.020	1.29 (1.10–1.52)	0.04
High	1175/1265	4	1.68 (1.25–2.27)	0.826	1.34 (1.09–1.65)	0.438	1.46 (1.13–1.89)	0.548		1.42 (1.17–1.73)	0.668	1.33 (1.16–1.53)	0.585
rs6983267 T>G (region3)													
All	12233/7582	17	1.44 (1.31–1.58)	0.065	1.19 (1.10–1.29)	0.065	1.26 (1.18–1.36)	0.473		1.54 (0.93–2.57)	0.000	1.19 (1.14–1.25)	0.187
publication bias (Begg’s test)			*P *= 0.308		*P *= 0.959		*P *= 0.072			*P *= 0.749		*P *= 0.303	
Ethnicity													
Caucasion	9879/5487	9	1.47 (1.33–1.64)	0.065	1.25 (1.07–1.46)	0.027	1.27 (1.17–1.38)	0.427		1.77 (0.81–3.87)	0.000	1.21 (1.15–1.27)	0.102
Asian	1922/1580	6	1.33 (1.08–1.62)	0.166	1.07 (0.92–1.24)	0.746	1.27 (1.06–1.52)	0.201		1.35 (1.04–1.75)	0.005	1.14 (1.03–1.25)	0.283
Source of control													
HB	4098/3021	11	1.68 (1.30–2.16)	0.038	1.17 (1.03–1.33)	0.141	1.38 (1.22–1.55)	0.223	1.43 (1.00–2.04)		0.000	1.24 (1.15–1.33)	0.092
PB	8135/4561	6	1.37 (1.22–1.54)	0.532	1.20 (1.09–1.33)	0.065	1.21 (1.10–1.32)	1.000	1.80 (0.63–5.11)		0.000	1.17 (1.10–1.24)	0.686
Score													
Low	1698/1516	7	1.79 (1.19–2.67)	0.009	1.11 (0.94–1.30)	0.120	1.43 (1.19–1.73)	0.069		1.34 (0.98–1.84)	0.002	1.28 (1.07–1.54)	0.015
High	10535/6066	10	1.42 (1.31–1.58)	0.547	1.22 (1.11–1.33)	0.121	1.24 (1.14–1.34)	0.986		1.66 (0.79–3.51)	0.000	1.19 (1.13–1.25)	0.839

HB, Hospital based; PB, Population based. OR, Odds ratio; CI, confidence interval.

**Table 3:** False-positive report probability values for associations between the risk of cancer and the frequency of genotypes of 8q24 variants.

8q24 SNP genotype	Crude OR (95%CI)	*P*^a^	statistical power^b^	Prior probability				
				0.250	0.100	0.010	0.001	0.000
**8q24-rs1447295 C**>**A**								
** All patients**								
** **Homozygous	1.39 (1.01–1.91)	<0.0001	0.993	**0.000**	**0.001**	**0.010**	**0.091**	0.502
** **Heterozygous	1.26 (1.09–1.45)	<0.0001	1.000	**0.000**	**0.001**	**0.010**	**0.091**	0.500
** **Recessive	0.51 (0.39–0.67)	<0.0001	0.896	**0.000**	**0.001**	**0.011**	**0.100**	0.527
** **Dominant	2.46 (1.89–3.20)	<0.0001	1.000	**0.000**	**0.001**	**0.010**	**0.091**	0.500
** **Allele comparision	1.23 (1.09–1.40)	<0.0001	1.000	**0.000**	**0.001**	**0.010**	**0.091**	0.500
** **Ethnicity-Caucasian								
** **Recessive	0.48 (0.31–0.74)	<0.0001	0.472	**0.001**	**0.002**	**0.021**	**0.175**	0.680
** **Dominant	4.15 (3.26–5.28)	<0.0001	1.000	**0.000**	**0.001**	**0.010**	**0.091**	0.500
** **Ethnicity-Asian								
** **Homozygous	1.87 (1.33–2.62)	<0.0001	0.059	**0.005**	**0.015**	**0.144**	0.630	0.944
** **Heterozygous	1.49 (1.30–1.70)	<0.0001	0.986	**0.000**	**0.001**	**0.010**	0.092	0.504
** **Recessive	0.38 (0.27–0.53)	<0.0001	0.028	**0.011**	**0.031**	0.262	0.781	0.973
** **Dominant	1.97 (1.47–2.62)	<0.0001	0.968	**0.000**	**0.001**	**0.010**	**0.094**	0.508
** **Allele comparision	1.44 (1.29–1.60)	<0.0001	1.000	**0.000**	**0.001**	**0.010**	**0.091**	0.500
** **Source of control-HB								
** **Recessive	0.52 (0.33–0.82)	0.0436	0.948	**0.121**	0.293	0.820	0.979	0.998
** **Dominant	2.07 (1.16–3.67)	<0.0001	1.000	**0.000**	**0.001**	**0.010**	**0.091**	0.500
** **Source of control-PB								
** **Homozygous	1.80 (1.52–2.13)	<0.0001	0.772	**0.000**	**0.001**	**0.013**	**0.115**	0.564
** **Heterozygous	1.38 (1.22–1.57)	<0.0001	1.000	**0.000**	**0.001**	**0.010**	**0.091**	0.500
** **Recessive	0.44 (0.37–0.52)	<0.0001	0.440	**0.001**	**0.002**	**0.022**	**0.185**	0.694
** **Dominant	3.22 (2.78–3.74)	<0.0001	1.000	**0.000**	**0.001**	**0.010**	**0.091**	0.500
** **Allele comparision	1.34 (1.21–1.50)	<0.0001	1.000	**0.000**	**0.001**	**0.010**	**0.091**	0.500
** **Score-Low								
** **Recessive	0.45 (0.25–0.81)	<0.0001	0.114	**0.003**	**0.008**	**0.080**	0.467	0.898
** **Dominant	1.79 (1.01–3.16)	<0.0001	0.995	**0.000**	**0.001**	**0.010**	**0.091**	0.501
** **Score-High								
** **Recessive	0.54 (0.39–0.74)	<0.0001	0.696	**0.000**	**0.001**	**0.014**	**0.126**	0.590
** **Dominant	3.05 (2.31–4.02)	<0.0001	1.000	**0.000**	**0.001**	**0.010**	**0.091**	0.500
** **Allele comparision	1.29 (1.14–1.46)	<0.0001	1.000	**0.000**	**0.001**	**0.010**	**0.091**	0.500
**8q24-rs16901979 C**>**A**								
** All patients**								
** **Homozygous	1.71 (1.36–2.16)	0.0663	0.995	**0.167**	0.375	0.868	0.985	0.999
** **Heterozygous	1.31 (1.12–1.53)	0.3859	1.000	0.537	0.776	0.974	0.997	1.000
** **Dominant	1.39 (1.20–1.61)	0.1506	0.999	0.312	0.576	0.937	0.993	0.999
** **Allele comparision	1.31 (1.18–1.46)	0.0524	1.000	0.136	0.320	0.838	0.981	0.998
** **Ethnicity-Asian								
** **Recessive	1.65 (1.13–2.41)	0.3859	0.908	0.560	0.793	0.977	0.998	1.000
** **Allele comparision	1.17 (1.00–1.38)	0.0394	0.999	**0.106**	0.262	0.796	0.975	0.997
** **Ethnicity-Africa-American								
** **Homozygous	1.91 (1.44–2.55)	<0.0001	0.902	**0.116**	0.282	0.812	0.978	0.998
** **Heterozygous	1.41 (1.10–1.80)	0.0028	0.837	**0.010**	**0.029**	0.249	0.770	0.971
** **Dominant	1.56 (1.24–1.96)	<0.0001	0.375	**0.001**	**0.002**	**0.026**	0.210	0.727
** **Allele comparision	1.40 (1.21–1.62)	<0.0001	0.371	**0.001**	**0.002**	**0.026**	0.212	0.730
** **Source of control-HB								
** **All patients								
** **Homozygous	1.71 (1.36–2.16)	0.0663	0.995	**0.167**	0.375	0.868	0.985	0.999
** **Heterozygous	1.31 (1.12–1.53)	0.3859	1.000	0.537	0.776	0.974	0.997	1.000
** **Dominant	1.39 (1.20–1.61)	0.1506	1.000	0.311	0.575	0.937	0.993	0.999
** **Allele comparision	1.31 (1.18–1.46)	0.0524	1.000	**0.136**	0.320	0.838	0.981	0.998
** **Score-Low								
** **Homozygous	1.75 (1.22–2.51)	0.6976	0.993	0.678	0.863	0.986	0.999	1.000
** **Recessive	1.49 (1.08–2.05)	0.7287	0.994	0.687	0.868	0.986	0.999	1.000
** **Allele comparision	1.29 (1.10–1.52)	0.6989	1.000	0.677	0.863	0.986	0.999	1.000
** **Score-High								
** **Homozygous	1.68 (1.25–2.27)	0.0297	0.883	**0.092**	0.232	0.769	0.971	0.997
** **Heterozygous	1.34 (1.09–1.65)	0.2149	1.000	0.392	0.659	0.955	0.995	1.000
** **Recessive	1.46 (1.13–1.89)	0.0551	0.926	**0.151**	0.349	0.855	0.983	0.998
** **Dominant	1.42 (1.17–1.73)	0.0546	0.999	**0.141**	0.330	0.844	0.982	0.998
** **Allele comparision	1.33 (1.16–1.53)	0.0131	1.000	**0.038**	**0.105**	0.565	0.929	0.992
**8q24-rs6983267 T**>**G**								
** All patients**								
** **Homozygous	1.44 (1.31–1.58)	<0.0001	1.000	**0.000**	**0.001**	**0.010**	**0.091**	0.500
** **Heterozygous	1.19 (1.10–1.29)	<0.0001	1.000	**0.000**	**0.001**	**0.010**	**0.091**	0.500
** **Recessive	1.26 (1.18–1.36)	<0.0001	1.000	**0.000**	**0.001**	**0.010**	**0.091**	0.500
** **Allele comparision	1.19 (1.14–1.25)	<0.0001	1.000	**0.000**	**0.001**	**0.010**	**0.091**	0.500
** **Ethnicity-Caucasian								
** **Homozygous	1.47 (1.33–1.64)	<0.0001	1.000	**0.000**	**0.001**	**0.010**	**0.091**	0.500
** **Heterozygous	1.25 (1.07–1.46)	0.0001	1.000	**0.000**	**0.001**	**0.010**	**0.091**	0.500
** **Recessive	1.27 (1.17–1.38)	<0.0001	1.000	**0.000**	**0.001**	**0.010**	**0.091**	0.500
** **Allele comparision	1.21 (1.15–1.27)	<0.0001	1.000	**0.000**	**0.001**	**0.010**	**0.091**	0.500
** **Ethnicity-Aasian								
** **Homozygous	1.33 (1.08–1.62)	0.0026	0.939	**0.008**	**0.024**	0.215	0.734	0.965
** **Recessive	1.27 (1.06–1.52)	0.0048	0.959	**0.015**	**0.043**	0.331	0.833	0.980
** **Dominant	1.35 (1.04–1.75)	0.0477	1.000	**0.125**	0.300	0.825	0.979	0.998
** **Allele comparision	1.14 (1.03–1.25)	0.0038	1.000	**0.011**	**0.033**	0.273	0.792	0.974
** **Source of control-HB								
** **Homozygous	1.68 (1.30–2.16)	0.001	1.000	**0.003**	**0.009**	**0.090**	0.500	0.909
** **Heterozygous	1.17 (1.03–1.33)	0.002	1.000	**0.006**	**0.018**	**0.165**	0.666	0.952
** **Recessive	1.38 (1.22–1.55)	0.0755	1.000	**0.185**	0.405	0.882	0.987	0.999
** **Dominant	1.43 (1.00–2.04)	0.0004	1.000	**0.001**	**0.004**	**0.038**	0.286	0.800
** **Allele comparision	1.24 (1.15–1.33)	0.0009	1.000	**0.003**	**0.008**	**0.082**	0.473	0.900
** **Source of control-PB								
** **Homozygous	1.37 (1.22–1.54)	<0.0001	1.000	**0.000**	**0.001**	**0.010**	**0.091**	0.500
** **Heterozygous	1.20 (1.09–1.33)	0.0021	1.000	**0.006**	**0.019**	**0.172**	0.677	0.955
** **Recessive	1.21 (1.10–1.32)	<0.0001	1.000	**0.006**	**0.019**	**0.172**	0.677	0.955
** **Allele comparision	1.17 (1.10–1.24)	<0.0001	1.000	**0.000**	**0.001**	**0.010**	**0.091**	0.500
** **Score-Low								
** **Homozygous	1.79 (1.19–2.67)	0.0008	0.871	**0.003**	**0.008**	**0.083**	0.478	0.902
** **Recessive	1.43 (1.19–1.73)	0.0007	0.863	**0.002**	**0.007**	**0.074**	0.448	0.890
** **Allele comparision	1.28 (1.07–1.54)	0.0016	1.000	**0.005**	**0.014**	**0.137**	0.615	0.941
** **Score-High								
** **Homozygous	1.42 (1.31–1.58)	<0.0001	1.000	**0.000**	**0.001**	**0.010**	**0.091**	0.500
** **Heterozygous	1.22 (1.11–1.33)	0.0003	1.000	**0.001**	**0.003**	**0.029**	0.231	0.710
** **Recessive	1.24 (1.14–1.34)	0.0034	1.000	**0.010**	**0.030**	0.252	0.773	0.971
** **Allele comparision	1.19 (1.13–1.25)	<0.0001	1.000	**0.000**	**0.001**	**0.010**	**0.091**	0.500

OR, Odds ratio; CI, Confidence interval. The results in false-positive report probability analysis were in bold, if the prior probability <0.2.

^a^Chi-square test was used to calculate the genotype frequency distributions.

^b^Statistical power was calculated using the number of observations in the subgroup and the OR and P values in this table.
